# Supporting elderly people with cognitive impairment during and after hospital stays with intersectoral care management: study protocol for a randomized controlled trial

**DOI:** 10.1186/s13063-019-3636-5

**Published:** 2019-08-30

**Authors:** Angela Nikelski, Armin Keller, Fanny Schumacher-Schönert, Terese Dehl, Jessica Laufer, Ulf Sauerbrey, Diana Wucherer, Adina Dreier-Wolfgramm, Bernhard Michalowsky, Ina Zwingmann, Horst Christian Vollmar, Wolfgang Hoffmann, Stefan H. Kreisel, Jochen René Thyrian

**Affiliations:** 1 Division of Geriatric Psychiatry, Evangelisches Klinikum Bethel, Bethesdaweg 12, 33617 Bielefeld, Germany; 2 German Center for Neurodegenerative Diseases (DZNE), site Rostock/Greifswald, Ellernholzstr. 1-2, 17489 Greifswald, Germany; 3 Department of Epidemiology and Community Health, Institute for Community Medicine, University Medicine Greifswald, Ellernholzstr. 1-2, 17489 Greifswald, Germany; 4 Institute of General Practice and Family Medicine, University Hospital Jena, Bachstr. 18, 07743 Jena, Germany; 5 Institute of General Practice and Family Medicine, Faculty of Medicine, Ruhr-University Bochum (RUB), Gebäude MA, Universitätsstraße 150, 44801 Bochum, Germany

**Keywords:** Discharge management, Health care, Dementia care, Collaborative care, Cognitive impairment, Care management, Case management

## Abstract

**Background:**

The sectorization of health-care systems leads to inefficient treatment, especially for elderly people with cognitive impairment. The transition from hospital care to primary care is insufficiently coordinated, and communication between health-care providers is often lacking. Consequences include a further deterioration of health, higher rates of hospital readmissions, and institutionalization. Models of collaborative care have shown their efficacy in primary care by improving patient-related outcomes. The main goal of this trial is to compare the effectiveness of a collaborative care model with usual care for people with cognitive impairment who have been admitted to a hospital for treatment due to a somatic illness. The aim of the intervention is to improve the continuity of treatment and care across the transition between the in-hospital and adjoining primary care sectors.

**Methods/design:**

The trial is a longitudinal multisite randomized controlled trial with two arms (care as usual and intersectoral care management). Inclusion criteria at the time of hospital admission due to a somatic illness are age 70+ years, cognitive impairment (Mini Mental State Examination, MMSE ≤26), living at home, and written informed consent. Each participant will have a baseline assessment at the hospital and two follow-up assessments at home (3 and 12 months after discharge). The estimated sample size is *n* = 398 people with cognitive inmpairement plus their respective informal caregivers (where available).

In the intersectoral care management group, specialized care managers will develop, implement, and monitor individualized treatment and care based on comprehensive assessments of the unmet needs of the patients and their informal caregivers. These assessments will occur at the hospital and in participants’ homes. Primary outcomes are (1) activities of daily living, (2) readmission to the hospital, and (3) institutionalization. Secondary outcomes include (a) frailty, (b) delirium, (c) quality of life, (d) cognitive status, (e) behavioral and psychological symptoms of dementia, (f) utilization of services, and (g) informal caregiver burden.

**Discussion:**

In the event of proving efficacy, this trial will deliver a proof of concept for implementation into routine care. The cost-effectiveness analyses as well as an independent process evaluation will increase the likelihood of meeting this goal. The trial will enable an in-depth analysis of mediating and moderating effects for different health outcomes at the interface between hospital care and primary care. By highlighting treatment and care, the study will provide insights into unmet needs at the time of hospital admission, and the opportunities and barriers to meeting those needs during the hospital stay and after discharge.

**Trial registration:**

ClinicalTrials.gov, NCT03359408; December 2, 2017.

**Electronic supplementary material:**

The online version of this article (10.1186/s13063-019-3636-5) contains supplementary material, which is available to authorized users.

## Background

The German health-care system is strongly sectorized with (a) outpatient treatment and care predominantly provided by general practitioners (GPs) and specialists in private practice, (b) inpatient treatment and care provided in hospitals, and (c) rehabilitation. While treatment and care within each of these sectors is generally of high quality, there is often a challenge to deliver continuous treatment and care across these sectors. Treatment pathways for people with chronic diseases or elderly people suffering from multiple morbidities and cognitive impairment often include frequent transitions between these sectors. However, in Germany, the boundaries between sectors are often rigid, rendering transitions between sectors a threat to treatment continuity and care coordination, which often results in unfavorable outcomes for patients. After this problem was identified and described in detail by the Advisory Council on the Assessment of Developments in the Health Care System in 2012 [1], a number of different remedies were proposed. Their impact, however, has been limited to date. This study addresses the lack of integrated cross-sectoral approaches to the challenges caused by the sectorization of the German health-care system.

In particular, there is a lack of sustainable management at the interface between in- and outpatient treatment. Treatment and care need to be oriented more toward the patients’ needs rather than the hospitals’ processes or the needs of specific diseases [2, 3]. There is evidence that patient involvement is positively associated with improved health behavior and treatment outcomes [4, 5]. In Germany, some discharge management is mandatory by law and must be available in each hospital [6]. It is financed by statutory health insurance and has just recently been specified and extended [7, 8]. However, the discharge management in routine care differs considerably from the expert standard proposed by, for example, the German Network for Quality Development in Nursing [9]. There should be more emphasis on an individualized needs assessment, continued care after discharge, and, most importantly, the inclusion of informal caregivers or relatives in the discharge process. For elderly people with cognitive impairment (PCI) in particular, it is well known that they have an increased need for professional and informal care at home. However, caregiving is burdensome [10, 11], and as such, informal caregivers require attention and support from health-care providers. Reducing the burden on caregivers is not only beneficial to the caregiver but also to the patient and—from a societal perspective—to the health-care system.

Current figures indicate that elderly people visit emergency units in hospitals more often than younger people do. In addition to their somatic illness, many of these elderly patients also face mental health problems. Approximately 50% of them have cognitive impairment, 27% suffer from delirium, 8–32% suffer from depressive symptoms, 21% suffer from apathy, and 9% suffer from agitation, aggression, or other symptoms [12]. In general, approximately half of the patients in hospitals are older than 65 years [13]. However, cognitive impairment is significantly underdiagnosed in hospitals. According to the German Federal Health Reporting Database (www.gbe-bund.de), 19,632,764 patients were treated in hospitals in Germany in 2014. Only 0.002% had a diagnosis of dementia [14]. However, an international review of dementia research suggests that prevalence rates range from 3.4% to 43.3%, depending on the age composition, study design, the setting, and the identification and screening criteria [15]. A nationwide survey of 1844 head nurses yielded an average point prevalence of 23% of PCI, independent of the specialty of the ward [16]. Accordingly, official data from the German Federal Health Reporting Database (www.gbe-bund.de) seem to underestimate the number of PCI and, consequently, the associated challenges for PCI, their informal caregivers, and the primary health-care providers. Current German primary data indicate that the point prevalence of PCI is close to 20% based on a screening test [17]. The point prevalence of dementing illnesses in German hospitals has been reported to be 18.4%. Delirium, most often due to dementia, was present in 5.1%. Only 60.0% had no cognitive impairment [18].

It is well established that cognitive impairment and the resulting behavioral and psychiatric symptoms pose a challenge for (a) the patient, relatives, and informal caregivers, (b) the treatment of the patient, (c) the hospital team caring for the patient and (d) the hospital setting in general. The behavioral and psychological symptoms of dementia are common in older PCI in hospitals and are associated with considerable distress in the nursing staff [19]. Admission to hospital is associated with cognitive problems or worsening of pre-existing cognitive problems, which increase the risk for readmission, institutionalization, and mortality [20–22]. These risks are aggravated by factors such as comorbidities, malnutrition, activities of daily living (ADL), depression, and other mental disorders.

Upon discharge from the hospital, these risks complicate the transition to primary care and increase the need for post-discharge support for patients living in their homes. However, the specific needs of these patients are not adequately addressed in Germany today: (a) postoperative treatment (by outpatient practitioners and therapists) and care arrangements are not sufficiently coordinated, (b) medical reports and patient data necessary for the continuation of treatment and care are communicated incompletely or delayed between hospitals and GPs, and (c) guidelines for intersectoral clinical pathways do not exist. These structural deficits, in summary, lead to insufficient treatment and care for many elderly PCI, cost-intensive unscheduled rehospitalizations, and premature institutionalization. Ultimately, both patients and health-care providers are often dissatisfied.

From a patient-centered perspective, one approach to overcoming these interface problems is collaborative case and care management. In Germany, the DelpHi-MV study (Dementia: Life- and Person-centred Help in Mecklenburg-Western Pomerania) [23, 24] tested the efficacy of a model of collaborative care of patients with dementia living in their own homes. The DelpHi-MV study is a GP-based cluster-randomized controlled intervention trial in primary-care settings that evaluated the efficacy of dementia care management [23]. The DelpHi-MV intervention improved relevant patient- and informal-caregiver-related outcomes [24]. Compared with care, the usual behavioral and psychiatric symptoms of dementia and informal caregiver burden were significantly decreased. Moreover, in the subgroup of patients who did not live alone, their quality of life increased. Patients with dementia receiving DelpHi care management had an increased chance of receiving antidementia drug treatment.

In a separate acceptance study, approximately 80% of the participating GPs indicated that care management as provided in the DelpHi-MV study should become a basic service in routine care [25]. Its further development for application in an intersectoral setting seems promising, since the intervention modules are well defined, the necessary qualification for the dementia care managers has been developed [26, 27], and a computerized intervention management tool has been programmed [28]. It is based on the principles of (i) individualized needs assessment, (ii) computer-supported development of a modular treatment and care plan, and (iii) implementation of this plan and the monitoring of its practicability and acceptance.

Based on the DelpHi experience, the objective of the Intersec-CM interventional trial is to test the efficacy of intersectoral care management for PCI around the time of discharge from the hospital back into primary care. The main hypotheses are as follows:

- PCI receiving intersectoral care management during the transition from hospital to their homes and into primary care will have better patient-oriented health outcomes than PCI receiving care as usual (CAU) at 3 and 12 months after discharge.
- PCI receiving intersectoral care management during the transition from hospital to home will have a lower frequency of unscheduled readmissions to hospital than PCI receiving CAU.
- PCI receiving intersectoral care management during the transition from hospital to home are less likely to be institutionalized 12 months after discharge from hospital than PCI receiving CAU.

## Methods/design

### Study design and sites

**Fig. 1:**
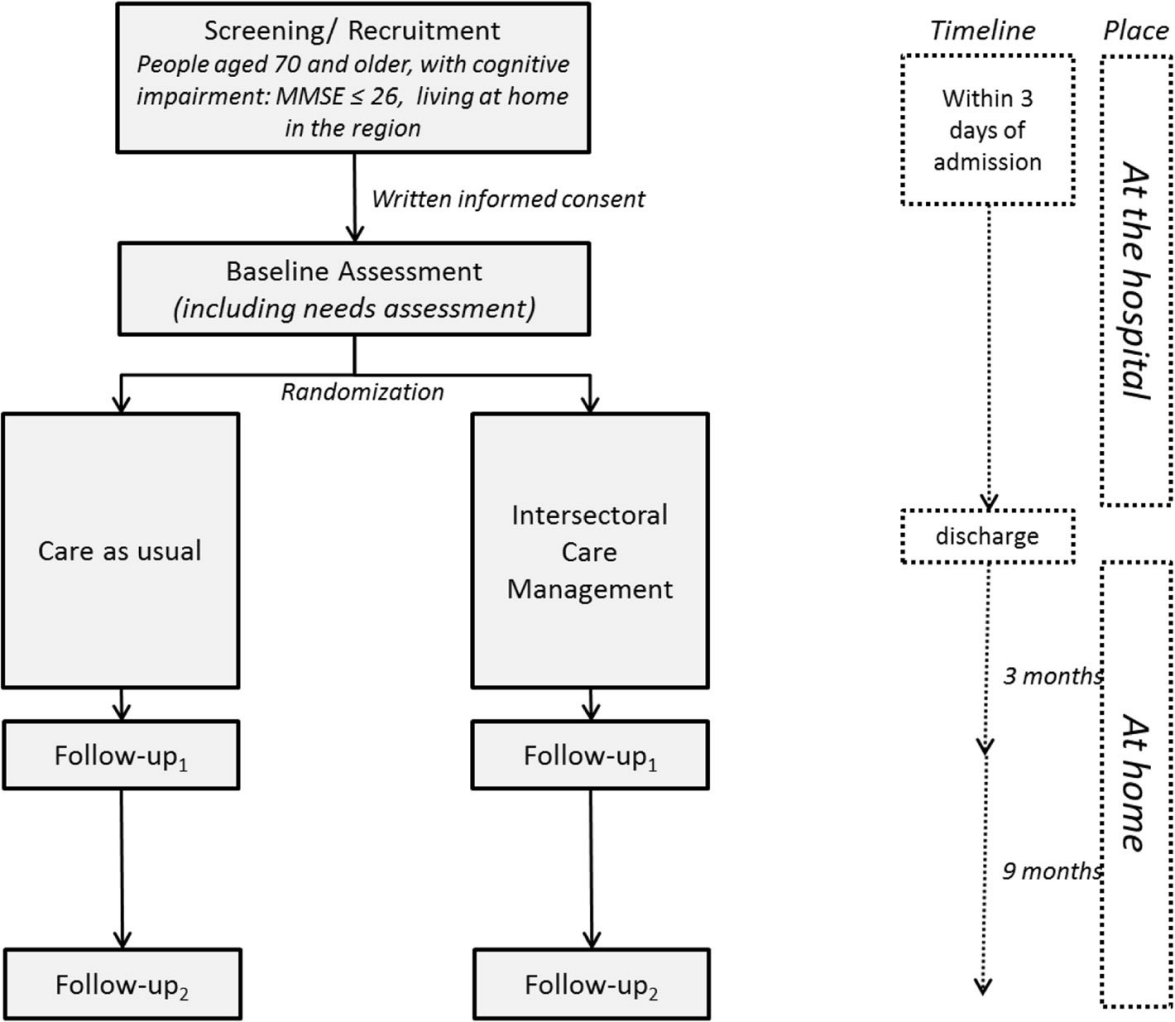
Flow chart for the intersec-CM trial. MMSE Mini Mental State Examination

This study is a multisite longitudinal randomized controlled intervention trial with two arms (intervention and CAU) and four time points of data assessment (screening, baseline, follow-up 1, and follow-up 2). The aim is to compare the effectiveness of an intervention (intersectoral care management designed to improve health and social outcomes of PCI in inpatient and ambulatory care) with CAU. Two hospitals are participating in this trial, the Evangelisches Klinikum Bethel in Bielefeld, North Rhine-Westphalia, and Greifswald Medical School, Mecklenburg-Western-Pomerania, with sites in Wolgast and Greifswald. The first patient was enrolled on 1 November 2018. Recruitment is planned for 12 months; thus, the last patient is expected to be enrolled on 31 October 2019. We expect the last follow-up assessment 13 months later, on 30 November 2020. The design of the study is summarized in Fig. 1. Ethical approval for this trial has been obtained from the ethics committee of Greifswald Medical School (registry number: BB 159/17) and the ethics committee of the Chamber of Physicians of Westphalia-Lippe (registry number: 2017–688-b-S). The trial is registered at ClinicalTrials.gov (NCT03359408).

### Study population and selection criteria

Elderly PCI admitted to a general hospital with somatic illnesses are eligible for the study. The inclusion criteria for these index participants were as follows: age 70+ with cognitive impairment detected with a standardized screening instrument (Mini Mental State Examination, MMSE ≤ 26) [29], having lived at home prior to the index admission, living in the catchment area of the hospital, and providing written informed consent (personally or by their legal guardian when necessary). The exclusion criteria are acute stroke as the primary reason for admission, terminal disease, or insufficient language skills.

Since the treatment and care of elderly PCI may be dependent on informal caregivers, index participants are asked to name their informal caregivers (e.g., a spouse, child, or friend). The informal caregiver is then invited as an independent study participant upon provision of written informed consent. While we put effort into recruiting this group, the participation of an informal caregiver is not a necessary requirement for the inclusion of the index participant.

### Intervention

The framework for the intervention is evidence-based dementia care management as conducted in the DelpHi-MV study [23, 24]. In principle, it consists of (i) a comprehensive assessment of the health and social status of the patient at the time of their admission to the hospital, (ii) a comprehensive needs assessment at the time of their admission to the hospital or shortly after, (iii) if applicable and available, an assessment of their informal caregiver’s health and burden, (iv) systematic written feedback to the treating hospital physician and nursing staff with recommendations for treatment and care for the patient after discharge (hospital information letter; these recommendations are based on predefined algorithms using the results of the assessment), (v) an assessment of the patient’s health at the time of discharge, (vi) a comprehensive needs assessment at the patient’s home immediately after discharge, (vii) if applicable and available, an assessment of the informal caregiver’s health and burden at the patient´s home, and (viii) support in meeting the patient’s needs as identified by the assessment.

The entire intervention delivery by specifically trained study nurses (case manager, social worker, or dementia care manager) [30] will be supported by a computerized tablet-based intervention management system for intersectoral care management. The system is a rule-based expert decision support system that matches individual patient characteristics to recommendations for treatment and care. The system supports the identification of a patient’s unmet needs, selects corresponding patient-specific interventions, and integrates these into an individualized intervention plan. The intervention management system has been proven to support the systematic identification of unmet needs, to improve the selection of specific intervention modules, and to address them systematically [25, 28].

The intervention used in this study does not, however, replace any provisions offered by other providers in the hospital or in ambulatory settings.

### Outcomes

The primary objective of the study is to evaluate the efficacy of intersectoral care management for elderly PCI admitted to hospital. Primary outcomes are ADL (which are a proxy for functionality after discharge), unscheduled readmissions, and premature institutionalization.

They are measured as follows:

- *Activities of daily living* will be assessed using the Bayer Activities of Daily Living Scale (B-ADL) [31], which consists of 25 items on everyday problems and challenges. The questionnaire targets community-dwelling patients who suffer mild cognitive impairment or mild-to-moderate dementia. It assesses the patient’s competence with general ADL and specific tasks in everyday life in the following domains: medication, hygiene, reading, conversation, telephoning, shopping, food preparation, handling money and financial affairs, using household appliances, transportation, leisure activities, everyday tasks that require unimpaired short- or long-term memory, and orientation in familiar and unfamiliar surroundings. The B-ADL also assesses nonspecific tasks requiring cognitive functions for the management of everyday life. These include remembering where to continue with an ongoing task after an interruption, doing two things at the same time, coping with unfamiliar or new situations that require the processing of new information, as well as the safety aspects of ADL and difficulties performing a task when under pressure.
- To assess *readmissions to hospital*, each participant will be asked how many times they have been hospitalized (unplanned and planned) within the last 12 months. This is one item in the Questionnaire on the Use of Medical and Non-Medical Services in Old Age (FIMA) [32], which is administered to assess utilization of health services. FIMA examines socioeconomic variables and other medical factors to determine health-related costs.
- *Institutionalization* will be assessed during the study, since the participants’ living situation will be checked at each assessment.

The secondary outcomes of this trial are not explicitly targeted by the intervention but were chosen as important moderating or mediating factors. Among those, we consider the following: (a) frailty, (b) delirium, (c) quality of life, (d) cognitive status, (e) neuropsychiatric symptoms, (f) utilization of health services, and (g) informal caregiver burden. We assume that (a), (b), and (f) are moderating factors and that (c), (d), (e), and (g) are mediating factors.

- Frailty will be measured using the Edmonton Frail Scale [33]. This is a reliable tool in geriatric medicine for assessing the frailty of older patients on the domains of cognition, general health status, functional independence, social support, medication use, nutrition, mood, continence and functional performance. Scores can range from 0 (not frail) to 17 (severe frailty).
- Delirium will be assessed using the Family Confusion Assessment Method [34]. It consists of up to seven items inquiring about the presence of different delirium-specific symptoms (yes or no) and one item about general mental health since the last visit (better, worse, or about the same). More information regarding the occurrence, stability, duration, frequency, and recency are asked when a symptom has been reported as present. An algorithm published by the developers is used to calculate a score for suggested delirium to be present (1) or not (0). This tool is a screening instrument and is not intended to provide a clinical diagnosis. Due to the economic and time constraints of this study, a detailed, guideline-oriented diagnosis of delirium will not be made.
- Quality of life of the PCI will be assessed using the five-dimension EuroQol Questionnaire for both the PCI and the caregiver [35]. This is a standardized instrument developed by the EuroQol Group to provide a simple generic measure of health in clinical and economic appraisals. Scores range from 0 (death) to 1 (best possible health).
- Cognitive status will be assessed using the MMSE [29]. This is a 30-point questionnaire designed to measure cognitive impairment. The questions are grouped into seven categories, each representing a different cognitive domain or function: orientation to time (5 points), orientation to place (5 points), registration of three words (3 points), attention and calculation (5 points), recall of three words (3 points), language (8 points), and visual construction (1 point). Scores range from 0 (lowest cognitive status) to 30 (cognitive status not impaired).
- Neuropsychiatric symptoms will be assessed using the Neuropsychiatric Inventory [36]. This is assessed using interview of the caregiver (when available) on 12 dimensions of neuropsychiatric behavior: delusions, hallucinations, agitation, dysphoria, anxiety, apathy, irritability, euphoria, disinhibition, aberrant motor behavior, night-time behavior disturbances, and appetite and eating abnormalities. Scores range from 0 (no neuropsychiatric symptoms) to 144 (severe symptoms, very often in all 12 dimensions).
- The utilization of health services will be assessed using the Resource Utilisation in Dementia Questionnaire [37] and FIMA [32]. The former will be used to assess the provision of informal care as well as the informal caregivers’ productivity losses. FIMA assesses the frequency of the utilization of medical and formal care services. Scores indicate whether a service is used (1) or not (0).
- Informal caregiver burden will be assessed using the seven-item (short) version of the Zarit Burden Inventory [38]. This is a caregiver self-report measure that examines the burden of caring associated with functional and behavioral impairments in social, psychological, and physiological contexts and at home. Scores range from 0 (no burden) to 28 (highest possible burden)

**Fig. 2:**
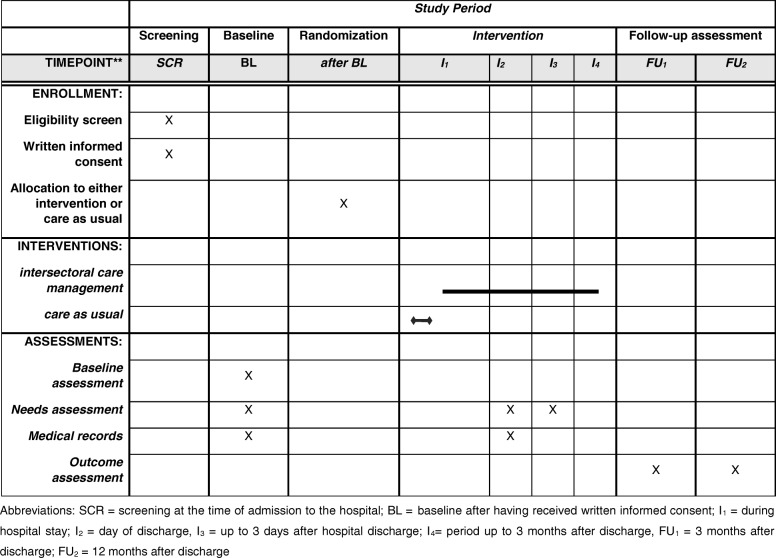
Schedule of enrollment, intervention, and assessments in accordance with the Standard Protocol Items: Recommendations for Interventional Trials (SPIRIT) guidelines. SCR screening at the time of admission to hospital, BL baseline after having provided written informed consent, *I*_1_ during hospital stay, *I*_2_ day of discharge, *I*_3_ up to 3 days after hospital discharge, *I*_4_ period up to 3 months after discharge, *FU*_1_ 3 months after discharge, *FU*_2_ 12 months after discharge

The data assessment tools for these dimensions will be chosen either based on their recommendation by the expert group of the Joint Programme Neurodegenerative Disease or because they are in common use in larger German trials such as IDEMUCK [39], DelpHi-MV [23, 40, 41], DemNet-D [42–45], and IDA [46]. These instruments have been validated, which will guarantee the validity of the results and comparability with German and international studies. An overview of the assessment points of the outcome measures is provided in Fig. 2.

### Sample size

The estimated enrollment for the study is *n* = 398 participants plus their respective informal caregivers (where available). This sample size is necessary in the context of a complex intervention, which could not be proven to be efficacious in a smaller cohort. A sample size of *n* = 199 persons per arm is needed to detect effects on ADL with a Cohen’s *d* = 0.25 [47]. We expect Cohen’s *d* = 0.25 based on the DelpHi-MV study [24], which was approximately the size of the effect of the intervention on patients’ ADL (from preliminary analyses, since ADL were not the primary outcome in this trial, but a secondary outcome). As the Intersec-CM intervention is adapted from dementia care management, this should give a good estimate of what effect will be obtained. In a comparison between the intervention and control groups at a significance level of *p* = 0.05 and a statistical power of 80%, the sample size needed to obtain the same effect size as in the DelpHi-MV study (*d* = 0.25) is *n* = 199 patients per arm. This calculation includes that outcomes expected to deliver stronger effects are sufficiently powered.

Because of the longitudinal design, the sample size will decrease over time for various reasons. Loss to follow-up due to death, migration, withdrawal of informed consent, and nonparticipation for logistical and organizational reasons will occur. Migration is unlikely to have a large effect because the 70+ year-old group tends to be geographically stable. If a participant is lost to follow-up, efforts will be made to locate and recontact them. Data on those lost to follow-up will be included in the main analysis. Withdrawal of initial consent will be documented wherever possible together with the reasons provided. We expect that loss to follow-up will be approximately 30%, so that we expect at least *n* = 279 participants will provide a completed data assessment at the second follow-up visit.

In summary, the sample size is sufficient to detect even moderate effects on the outcomes. International studies are not readily comparable to the specificities of the German health-care system, and since this is the first randomized controlled trial addressing these research questions in Germany [48], we cannot precisely calculate our sample size based on previous studies and do rely to some extent on assumptions. The trial will aim to reach the sample size calculated and will be prolonged if recruitment takes longer than expected.

### Study procedure

Target patients will be screened for cognitive impairment by study staff at the time of hospital admission or shortly after when their clinical status allows for this. The screening procedure consists of assessments to estimate cognitive impairment (including delirium) and its severity, including the 4AT [49], the Richmond Agitation-Sedation Scale [50], and MMSE [29].

Those people meeting all the inclusion criteria will be informed personally and in writing about the intersec-CM trial. They will be invited to participate, and upon providing written informed consent, a baseline assessment will be conducted. The baseline assessment is based on the patients’ hospital records (sociodemographic data, ICD-10 diagnoses, and medication history) and a personal structured interview. The interview uses established and valid instruments to assess ADL (Barthel Index [51]), frailty (Edmonton Frailty Scale [33]), nutrition (screening items of the Mini Nutritional Assessment [52]), mobility (Hierarchical Assessment of Balance and Mobility [53]), pain (screening items), sleep quality (screening items from the Pittsburgh Sleep Quality Index [54]), and depression (screening items). The interview includes a comprehensive needs assessment based on the Camberwell Assessment of Need for the Elderly questionnaire [55] and an adapted version of the DelpHi-MV needs assessment [26, 40].

After the baseline assessment, each individual will be randomized using computerized permuted blocks into either the intervention or CAU group. This method ensures that the intervention and CAU participants are distributed evenly in a ratio of 1:1. Allocation to either the intervention or CAU is conducted at the study center after the baseline assessment so that the study staff are blind to the allocation during the initial assessment and cannot influence the allocation. Full blinding will not be possible once the intervention has started due to the type of intervention.

Immediately following randomization, standardized and systematic feedback based on clinically relevant parameters ascertained in the baseline assessment will be issued to the treating hospital staff (hospital information letter) in both groups. The letter includes assessment data. For the intervention group, it additionally includes recommendations for treatment and care for the discharge plan and for the preparation of ambulatory services, medication, social integration, and medical treatment after discharge. In both groups, the participant’s medical status at the time of discharge will be documented in their record. Patients in the intervention group will be contacted immediately after discharge, if possible in person, to continue the delivery of the intervention as outlined above. Otherwise, telephone contact is initiated with the intention to plan a timely in-person visit or visits by the specifically trained study nurses.

Follow-up assessments of all outcome measures for both groups will be conducted 3 months and 12 months after discharge in structured personal interviews, preferably at the participant’s home. The place and time of the follow-up assessments are chosen for the highest possible convenience of the participants.

### Data collection

Specially trained study staff will collect the data needed for the trial in computer-assisted face-to-face interviews and from patient records. All interviews will be standardized using validated and reliable questionnaires. To reduce interviewer bias, study staff will be retrained and supervised weekly to ensure the optimum degree of comparability. The source of information in the interviews will predominantly be the participant with cognitive impairment. However, due to the cognitive impairment, the validity of the information obtained may be compromised. The informal caregiver—if available—will validate facts (such as sociodemographic status and ADL) and fill in gaps in the patient’s answers. The source of each data item will be documented and controlled for.

Data will be stored on a tablet-based computer at the time of assessment. This allows for plausibility checks at the time of assessment and will avoid the errors that would otherwise result from transferring the data from written paper notes to a computer. Data collection will take place in the hospital (at screening and at baseline) and at the participants’ home (at the first and second follow-ups).

### Data analysis

#### Statistical analysis

The statistical analyses will be calculated on an *intention-to-treat* basis to reduce the impact of dropouts during the follow-up. These will include all individuals with baseline values of the outcome variables. Missing follow-up values will be imputed. To check whether systematic dropouts during the baseline assessment influenced the results, we will run multivariable logistic regressions with dropout as a dichotomous outcome (yes or no). The study group, sociodemographic variables, and clinical parameters assessed during screening (such as MMSE and the 4A test) will be included as predictors. These analyses will be performed for (1) all dropouts, (2) dropouts due to death, and (3) dropouts due to withdrawal of informed consent. To describe the study sample, appropriate summary statistics such as the mean, median, minimum, and maximum for continuous variables and frequencies and percentages for categorical data will be used.

The primary analyses will use separate generalized linear models to test intervention efficacy. The main outcome variables at follow-up 1 (3 months after discharge) and follow-up 2 (12 months after discharge) will be the dependent variables. The model specification will correspond to the scale level of the outcome variable under investigation. B-ADL will be assessed with a linear mixed regression, while for readmissions into hospital and institutionalization, logistic models will be used. The models will be adjusted for age, sex, and living situation of the patients. The study group is the predictor of interest (CAU vs. intervention). To account for the stochastic dependency of patients treated in the different hospitals (Bielefeld and Greifswald), study center will be included as a random effect variable. The baseline value of the outcome variable will be included as a covariate to reduce residual variance and to account for inter-individual variance at baseline. A positive intervention effect is defined as a significant regression coefficient (one-sided test) of the study group variable. Missing data will be imputed by multiple imputations via chained equations, stratified by study group. The overall alpha of the different primary outcome analyses will be adjusted using the Bonferroni–Holm procedure [56].

For the secondary analyses, sociodemographic variables (age under 80 or above 80), sex (man or woman), living situation (alone or not alone), and clinical parameters such as cognitive status, delirium, and frailty will be included. These variables either showed an impact in the DelpHi-MV study [24, 57] or are expected to moderate the health trajectory of elderly people in general. The inclusion of sociodemographic and clinical parameters should result in decreased residual variance and enhanced statistical power. To improve the quality of the regression models, possible interaction effects will be analyzed for study group, age group, living situation, and clinical parameters.

Economic evaluations will be conducted using methods that are consistent with those of published methodological guidelines for economic evaluations [58]. Health-care costs per patient will be calculated for a retrospective period of 3 months (follow-up 1) and 1 year (follow-up 2) from a public payers and societal perspective using published unit costs [59]. To analyze differences in quality-adjusted life years (QALYs) and costs, a linear mixed regression model will be used. The incremental cost-effectiveness ratio will be calculated using the incremental cost per QALY gained by the intervention compared with usual care. We will calculate the probability of the intervention being cost-effective at a wide range of willingness-to-pay margins using nonparametric bootstrapping to handle the sample uncertainty [60–62].

### Quality assurance and safety

Several methods are used to ensure the quality of all dimensions measured in this trial. A scientific advisory board will be established, comprising experts in the field, which will meet twice during the trial. The first meeting was held in January 2018. The design of the trial was critically discussed and fine-tuned. A second meeting will be held in 2020 to advise on the scientific analysis and to discuss the initial results.

A trial steering committee has been established, comprising all research partners. This committee meets at least every 6 months to monitor the trial. It evaluates whether the study is adhering to the timeline, whether the work packages are being sufficiently addressed, and whether all milestones are being met. If difficulties in meeting the requirements are encountered, this committee will propose solutions at the management level. Any proposed alterations to the study design will be discussed with the funding agency.

To ensure the quality of intervention delivery, regular supervision of the study staff will monitor intervention delivery and fine-tune methods, skills, and knowledge. To guarantee a high degree of standardization of the intervention, a computer-assisted intervention management system will be used.

The processing and storage of the data must comply with legal standards for data privacy, protection, and safety. The data management complies with the current version of the data protection guidelines of the Institute for Community Medicine, Greifswald. These guidelines have been approved by the data safety and freedom of information officer of Mecklenburg-Western Pomerania. They contain specific regulations regarding cooperation with the German Center for Neurodegenerative Diseases at Rostock/Greifswald and other partners. The guidelines include data security as well as data protection measures. For example, aspects such as management of informed consent, restricted access to data that can be used to identify patients and medical data, pseudonymization for data analysis and dissemination, disposal of data that can be used to identify patients, and IT security measures are addressed.

### Limitations

There are several limitations of this trial, which might be a threat to the reliability, validity, or generalizability of the results.

1. All subjects in the study are staying in hospital due to an acute illness or planned treatment that is not necessarily associated or connected with cognitive impairment. This causes heterogeneity in the index illnesses, which might decrease comparability between subjects. However, there is an objective, reliable, and valid inclusion criteria for each participant, so that conclusions can be drawn for this sample. Also, the study is a close-to-routine-care trial and since the complex intervention aims at the whole health-care situation with consideration of comorbidity, this will not decrease the usefulness of our results.
2. Adherence to the study protocol might be difficult because the trial is conducted in routine care rather than in an experimental setting. This is a trade-off between evaluating the effect purely due to the intervention and the effect under close-to-routine conditions. We chose the second aim since it raises the chances of and gives more guidance to the implementation (if the intervention is successful).
3. Cognitive impairment might decrease the validity of the information given by the participants. However, some measures (e.g., quality of life) must be provided directly by the participant, since they are subjective. Other information can and will be checked by the study assistants, dependent on the cognitive status of the participant (like demographic data). We do include caregivers in the study and access patient records to achieve the best possible valid information. Furthermore, we will include cognitive status as a mediating factor in our analyses.

## Discussion

The relevance of this study for old and very old people is high. Intersec-CM addresses: (a) relevant problems in the health-care system (sectorization of in-hospital and primary care) and (b) inadequate treatment and care for acutely ill patients with cognitive impairment in the transition between hospital and primary care as a highly prevalent target group. It makes use of evidence-based methods with relevant outcomes for elderly patients (quality of life and ADL) and relevant outcomes to the overall health of the target group.

We expect that the trial will add scientific evidence to improve the treatment and care of PCI at the interface between hospital and primary care. We assume that the adapted dementia care management will improve patient-oriented outcomes as well as system-relevant outcomes. In the event of proving efficacy, this trial will deliver a proof of concept for implementation into routine care and—ideally—will improve the current health-care system. The results expected from this trial could facilitate the implementation of intersectoral care management systematically on a larger scale. Cost-effectiveness analyses as well as an independent mixed-methods process evaluation (which will be described in more detail elsewhere) increase the likelihood of meeting these goals.

Scientifically, the trial allows an in-depth analysis of mediating and moderating effects for different health outcomes at the interface between hospital and primary care. We expect frailty to be a risk factor for worse health outcomes over time. Additionally, we will add to the knowledge base of the trajectory of cognitive status and delirium from hospital stays to primary care. Identifying risk factors will help to improve treatment and care by allowing these to be targeted in future interventions or by making changes to the system.

By highlighting treatment and care, our study will provide insights into unmet needs at the time of hospital admission, and the opportunities and barriers to meeting those needs during the hospital stay or shortly after. There will be descriptive analyses of common needs that emerge directly after discharge and an estimate of which of these needs can be met immediately. Recommendations for how intersectoral care management can be implemented in the current systems will be developed and promoted.

Informal caregivers will be actively involved in all phases of the study. It is now common knowledge that they play an important role in dementia care. Our trial will provide descriptive details of which informal caregivers are involved, at what times, to what extent, and how. We will add descriptive knowledge about the informal caregivers, their social situation, their own (unmet) needs, whether they may benefit from the intervention, and if yes, how exactly. This knowledge may influence how informal caregivers are systematically involved (and supported) in treatment and care at the interface between hospital and primary care and sustainably thereafter.

Ultimately, the results will not be limited to PCI but will extend to elderly people transitioning between in-hospital and primary care in general Additional file 1.

### Trials status

The trial is currently recruiting. The first participant was enrolled on 1 November 2018. By 22 July 2019, 236 participants had been enrolled. The expected end of recruitment is 31 October 2019. The current protocol is version 1.0, dated 20 February 2019.

## Additional file

Additional file 1: SPIRIT 2013 checklist: recommended items to address in a clinical trial protocol and related documents. (DOC 121 kb)

## Data Availability

There is no plan to provide public access to the data.

## Abbreviations

ADL: Activities of daily living
B-ADL: Bayer Activities of Daily Living Scale
CAU: Care as usual
DelpHi-MV: Dementia: Life- and Person-centred Help in Mecklenburg-Western Pomerania
FIMA: Questionnaire on the Use of Medical and Non-Medical Services in Old Age
GP: General physician or general practitioner
Intersec-CM: Intersectoral Care Management Trial
MMSE: Mini Mental State Examination
PCI: People with cognitive impairment
QALY: Quality-adjusted life years
